# An open-access global database of meta-analyses investigating yield and biodiversity responses to different management practices

**DOI:** 10.1016/j.dib.2023.109696

**Published:** 2023-10-22

**Authors:** Elina Takola, Jonathan Bonfanti, Ralf Seppelt, Michael Beckmann

**Affiliations:** a Department of Computational Landscape Ecology, UFZ—Helmholtz Centre for Environmental Research, Permoserstrasse 15, Leipzig, 04318, Germany; b Institute of Geoscience & Geography, Martin-Luther-University Halle-Wittenberg, Halle (Saale), Germany; c Department of Agriculture, Ecotrophology and Landscape Development, Anhalt University of Applied Sciences, 06406 Bernburg, Germany; d German Centre for Integrative Biodiversity Research (iDiv), Halle-Jena-Leipzig, Puschstrasse 4, 04103 Leipzig, Germany; e Eco&Sols, Univ Montpellier, CIRAD, INRAE, Institut Agro, IRD, Montpellier, France

**Keywords:** Organic, Diversification, Nutrient, Biomass, Grazing, Synthesis, Species richness, Tillage

## Abstract

We here present a database of evidence on the impact of agricultural management practices on biodiversity and yield. This database is the result of a systematic literature review, that aimed to identify meta-analyses that use as their response variables any measure of biodiversity and yield. After screening more than 1,086 titles and abstracts, we identified 33 relevant meta-analyses, from which we extracted the overall estimates, the subgroup estimates as well as all information related to them (effect size metric, taxonomic group, crop type etc.). We also extracted information relative to the empirical studies used for each meta-analysis and recorded the countries in which they took place and assessed the quality of each meta-analysis. Our dataset is publicly accessible and can be used for conducting second-order meta-analyses on the effect of management measures on species richness, taxon abundance, biomass and yields. It can also be used to create evidence maps on agriculture-related questions.

Specifications Table

**Table:** 

Subject	**Agricultural Sciences / Agronomy and Crop Science**
Specific subject area	Synthesis of evidence and spatial information to explore the impact of various agricultural measures.
Data format	Raw
Type of data	Table
Data collection	Data were collected through a systematic literature review. We screened titles and abstracts from different sources. The database consists of the effect sizes and relevant information of these meta-analyses.
Data source location	Global
Data accessibility	Repository name: Open Science Framework Direct URL to data: https://osf.io/7s9r4/

## 1 Value of the Data

• Hundreds of empirical studies are published every year on the effect of agricultural management on biodiversity and yield. This big amount of information requires frequent syntheses, such as meta-analyses and systematic reviews on the effectiveness of each practice. We here present a collection of meta-analyses that examine the effects of multiple management practices on biodiversity and yield. We aim to facilitate the comparison across management practices, for example to answer whether organic farming increases biodiversity more than diversification. By making our data publicly accessible in the Open Science Framework, we aim to ensure their findability and accessibility. The provided files are in csv and txt format and can be easily handled and combined with other data. In addition, we describe the procedure we followed to compile this dataset and the R scripts used for the tables and figures. By exploring and analyzing these datasets, users can identify knowledge gaps and create evidence maps.
• Scientists in the fields of agronomy, landscape ecology and other related fields can reuse and/or combine our datasets with other data, in order to draw general conclusions about the effects of management practices on both biodiversity and yield. In addition, practitioners and decision-makers can use our data too, in order to obtain a comprehensive overview of current evidence.
• The data can be used to identify the management practices that ensure win-win scenarios in agricultural management. Indicatively, the data can be used in a mixed-effects model that will unveil the relative importance of variables such as crop type, functional group etc. The weighing of the different meta-analyses based on their quality can be done by using the sample size, measures of uncertainty (standard error, standard deviation and confidence intervals) and quality (from the “Quality assessment” table). Evidence maps might consist of world maps with the amount of evidence in each country, or even the effect sizes.

## 2 Data Description

**Fig. 1:**
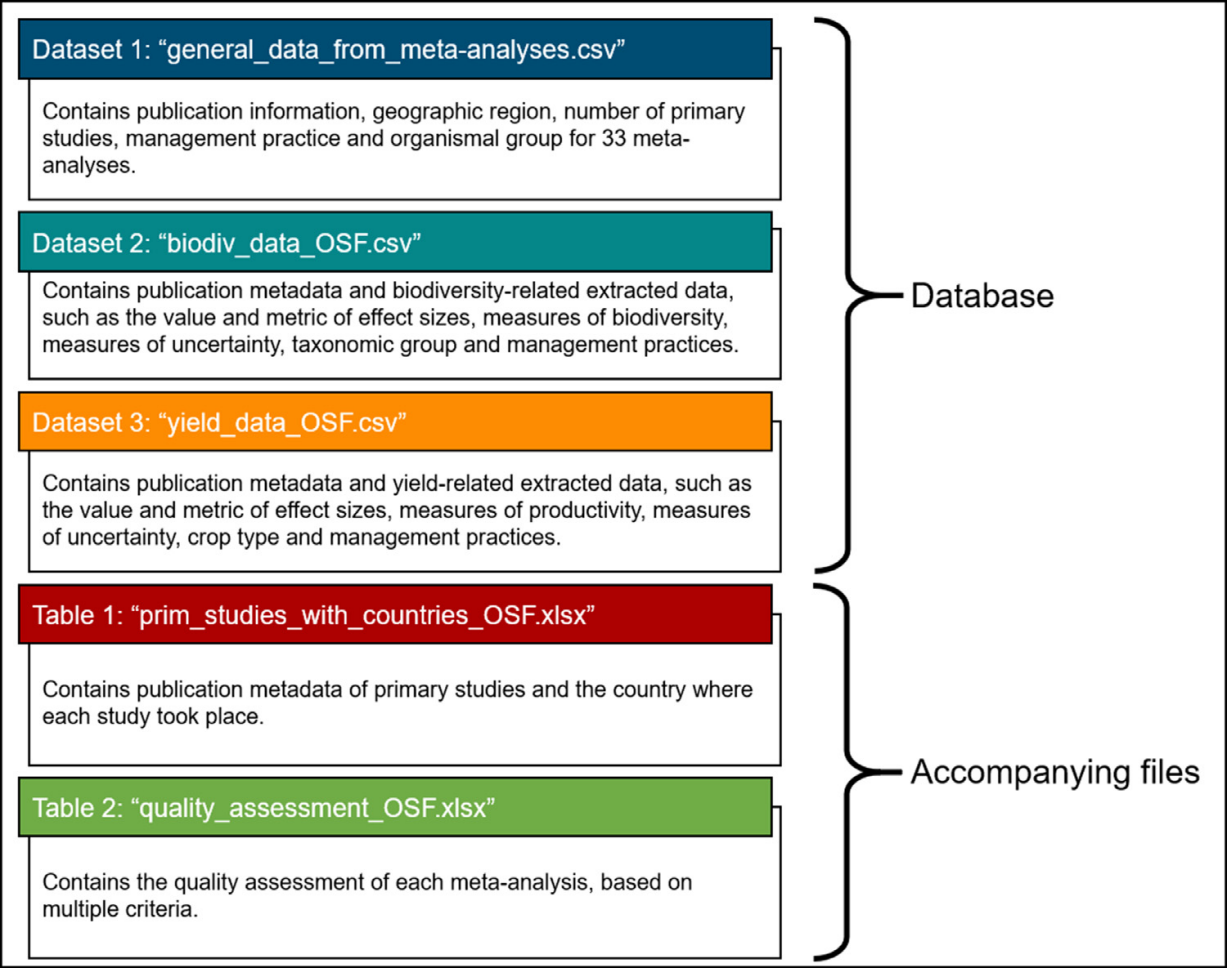
The structure of the database in the OSF folder, along with the filenames and their content.

We compiled multiple datasets using information extracted from the 33 meta-analyses, which were identified through a systematic literature review. Our database consists of three datasets (Fig. 1): one containing general information (Dataset 1), one containing the effect sizes of biodiversity (Dataset 2) and another one containing the effect sizes of yield (Dataset 3). The database has two accompanying tables: a list of primary studies (n = 1,967), including also the country of each study and a table with the quality assessment of each meta-analysis.

### 2.1 General information table

Dataset 1 consists of general information regarding the meta-analyses. Each row represents one meta-analysis and the columns correspond to metadata, described in Table 1. Notably, 8 meta-analyses found publication bias in their data overall or at least in one subgroup, whereas 14 studies did not mention a test for publication bias.

**Table 1:** Description of column names and abbreviations of the database “general_data_from_meta-analyses.csv”.

Column names	Description	Type of variable
ID	Unique identifier for each meta-analysis	Alphanumeric unique identifier
Title	The title of the meta-analysis	Text
Authors	The last name of the first author and year	Alphanumeric unique identifier
Year	Publication year of each meta-analysis	Numeric
DOI	Digital Object Identifier	URL
Number of primary studies	How many primary studies were used in each meta-analysis?	Numeric
Publication bias	Was there any sign of publication bias in the overall dataset or in any subgroup?	Yes/No
Geographic region	Did the meta-analysis focus on a specific region?	Text
Management practice raw	Which management practice was studied (as mentioned in the study)?	Text
Organismal group	Did the meta-analysis focus on an organismal group?	Text

The number of primary studies ranged from 26 to 328, while, although we did not set any filter related to time, the publication year of the meta-analyses ranged from 2011 to 2022. Most meta-analyses (n = 22) were global and 2 were focusing on Africa, 5 in China, 3 in Europe, 1 in North America and 1 meta-analysis was specific in Brazil. Regarding the organismal groups, 19 meta-analyses were considering all organismal groups, 7 were about plants and pollinators, microbes, herbivores, fungi, earthworms, biocontrol species and bacteria were the main study groups in 1 meta-analysis each.

The meta-analyses included in our dataset used different effect size metrics to answer the question of how a particular management practice affects biodiversity and yield. The effect size metrics present in our database are:

(i) Standardized mean difference, N = 1 study [1]
(ii) (Log) response ratio (weighted or unweighted), N = 21 studies [2], [3], [4], [5], [6], [7], [8], [9], [10], [11], [12], [13], [14], [15], [16], [17], [18], [19], [20], [21], [22]
(iii) Percentage change, N = 11 studies [22], [23], [24], [25], [26], [27]
(iv) Hedges G or D (weighted or unweighted), N = 10 studies [28], [29], [30], [31], [32]
(v) Cohens D, N = 1 study [33]

We created one dataset for biodiversity and another one for yield, in order to facilitate interoperability and achieve a more flexible format. The two datasets can be merged, depending on the research question, by joining them based on the columns they have in common.

**Fig. 2:**
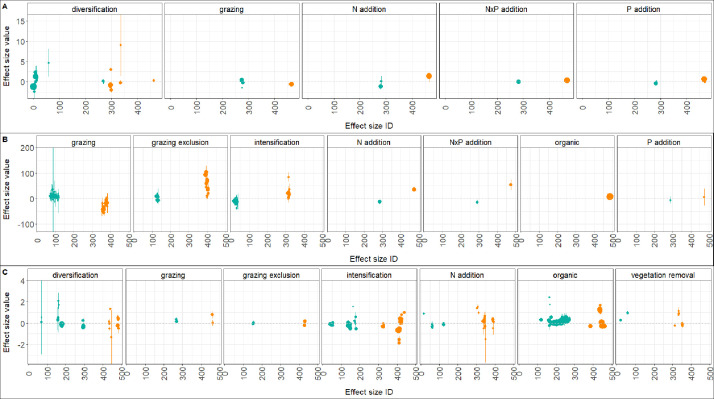
Effect sizes of biodiversity (teal) and yield (orange) grouped by effect size metric (A: Hedges, B: Percentage change, C: log response ratio) and management practice, for groups with more than 6 effect sizes. Points and vertical lines correspond to the value of the effect sizes (*y* axes). Numbers on the *x* axes are random identifiers of each effect size. The size of the dots is proportional to the sample size of each effect size. Horizontal dashed grey lines indicate a zero intercept. (For interpretation of the references to color in this figure legend, the reader is referred to the web version of this article.)

A comprehensive visualization of the effect sizes included in both datasets is given in Fig. 2, categorized by effect size metric and management practice. We separated the effect size by metric because each metric has a different numeric range (hence the differences in the y axes).

### 2.2 Biodiversity dataset

Dataset 2 consists of the meta-analytic estimates of the impact of management practices on various biodiversity measures (species richness, Shannon index etc.). Each row is one estimate and refers to either the overall meta-analytic mean or a subgroup estimate. If the value refers to a specific taxonomic group, the name of the subgroup is indicated in the column “Taxonomic group”. The column names and all abbreviations included in Dataset 2 (“biodiv_data_OSF.csv”) are described below.

• “ES_ID” is a unique identifier of each effect size.
• “StudyID” is a unique identifier of each meta-analysis.
• “Title”, “Authors”, “Year”, “DOI” are the publication information of each meta-analysis.
• “Biodiv_ES” is the value of the effect size.
• “Biodiv_ESMetric” is the effect size metric: Hedges’ D (HedgesD), Hedges’ G (HedgesG), Cohens’ D (CohensD), Response ratio (RR), Log response ratio (LRR), Standardized mean difference (StdMeanDiff), Percentage change.
• “Biodiv_measure” is the measure of biodiversity: Abundance (Ab), species richness (SR), Shannon's Index (Sh), diversity (Diversity), Shannon-Wiener Index (ShW), Pielou's Index (Pielou), species evenness (Evenness), biodiversity, biocontrol.
• “Biodiv_sample_size” is the sample size of each effect size.
• “Biodiv_CI_lower” is the lower bound of the confidence interval of each effect size.
• “Biodiv_CI_upper” is the upper bound of the confidence interval of each effect size.
• “Taxonomic_group” is the taxonomic group, whenever the effect size was referring to a specific group. If the effect size referred to all the species pooled, it was indicated with “all”.
• “Management_grouped” is the management practice.
• “SE” is the standard error.
• “SD” is the standard deviation.
• “ConfidenceInterval” is the type of confidence interval (90 % or 95 %).

**Fig. 3:**
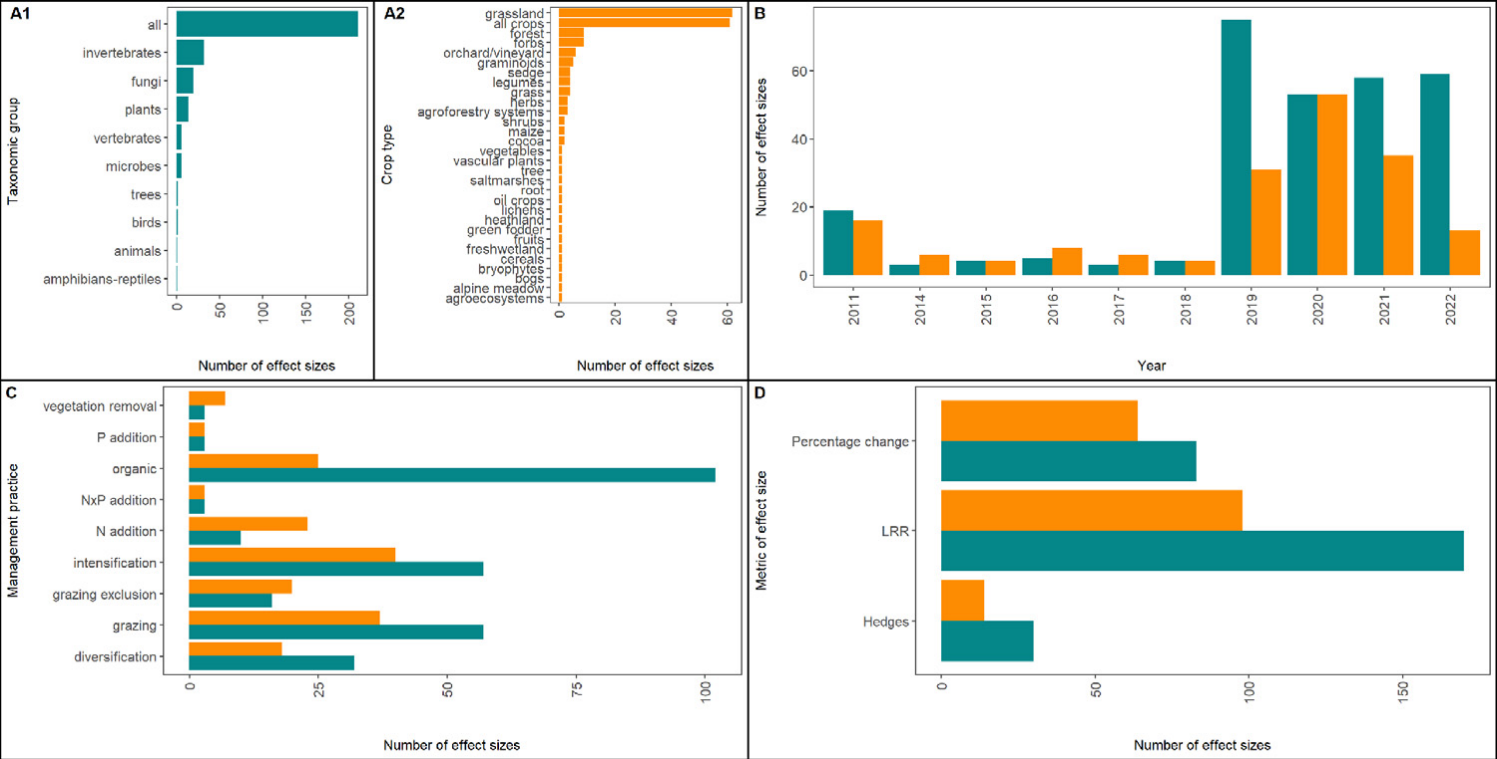
Barplots with number of effect sizes per subgroup for biodiversity (A1) and yield (A2), year (B) management practices (C) and effect size metric (D).

In total, we extracted 302 estimates from the 33 meta-analyses. This dataset represents the responses of 9 taxonomic groups to 7 management practices over a period of 11 years (Fig. 3).

### 2.3 Yield dataset

Dataset 3 consists of the meta-analytic estimates of the impact of management practices on various yield measures (yield, biomass etc.). Each row is one estimate and refers to either the overall meta-analytic mean or a subgroup estimate (name of subgroup is indicated in the column “Subgroup”).

The column names and all abbreviations included in Dataset 3 (“yield_data_OSF.csv”) are described below.

• “ES_ID” is a unique identifier of each effect size.
• “StudyID” is a unique identifier of each meta-analysis.
• “Title”, “Authors”, “Year”, “DOI” are the publication information of each meta-analysis.
• “Prod_ES” is the value of the effect size.
• “Prod_ESMetric” is the effect size metric: Hedges’ D (HedgesD), weighted Hedges’ D (HedgesD++), Hedges’ G (HedgesG), Cohens’ D (CohensD), Response ratio (RR), weighted response ratio (RR++), Log response ratio (LRR), Standardized mean difference (StdMeanDiff), Percentage change.
• “Prod_measure” is the measure of yield: Abundance, biomass, composite measures, cover, density, food, productivity, timber, yield.
• “Prod_sample_size” is the sample size of each effect size.
• “Prod_CI_lower” is the lower bound of the confidence interval of each effect size.
• “Prod _CI_upper” is the upper bound of the confidence interval of each effect size.
• “Subgroup” is the taxonomic group, whenever the effect size was referring to a specific group. If the effect size referred to all the species pooled, it was indicated with “all”.
• “Crop type” is the type of crop related to each effect size: all crops (when the effect size referred to all the crop types pooled), grassland, graminoids, forbs, forest, green fodder, agroecosystems, alpine meadow, shrubs, cocoa, herbs, heathland, fresh wetland, saltmarshes, bogs, bryophytes, lichens, tree, orchard/vineyard, vascular plants, grass, sedge, legumes, agroforestry systems, cereals, fruits, oil crops, root vegetables, grapes, maize
• “Management_grouped” is the management practice.
• “SE” is the standard error.
• “SD” is the standard deviation.
• “ConfidenceInterval” is the type of confidence interval (90 % or 95 %).
• “Notes” is a column containing notes related to each effect size.

Interestingly, two meta-analyses used the 90 % benchmark instead of 95 % for the confidence intervals of the effect sizes. In total, we extracted 205 estimates from the 33 meta-analyses. The data represent the responses of 30 crop types to 8 management practices over a period of 11 years (Fig. 3).

### 2.4 Primary studies

**Fig. 4:**
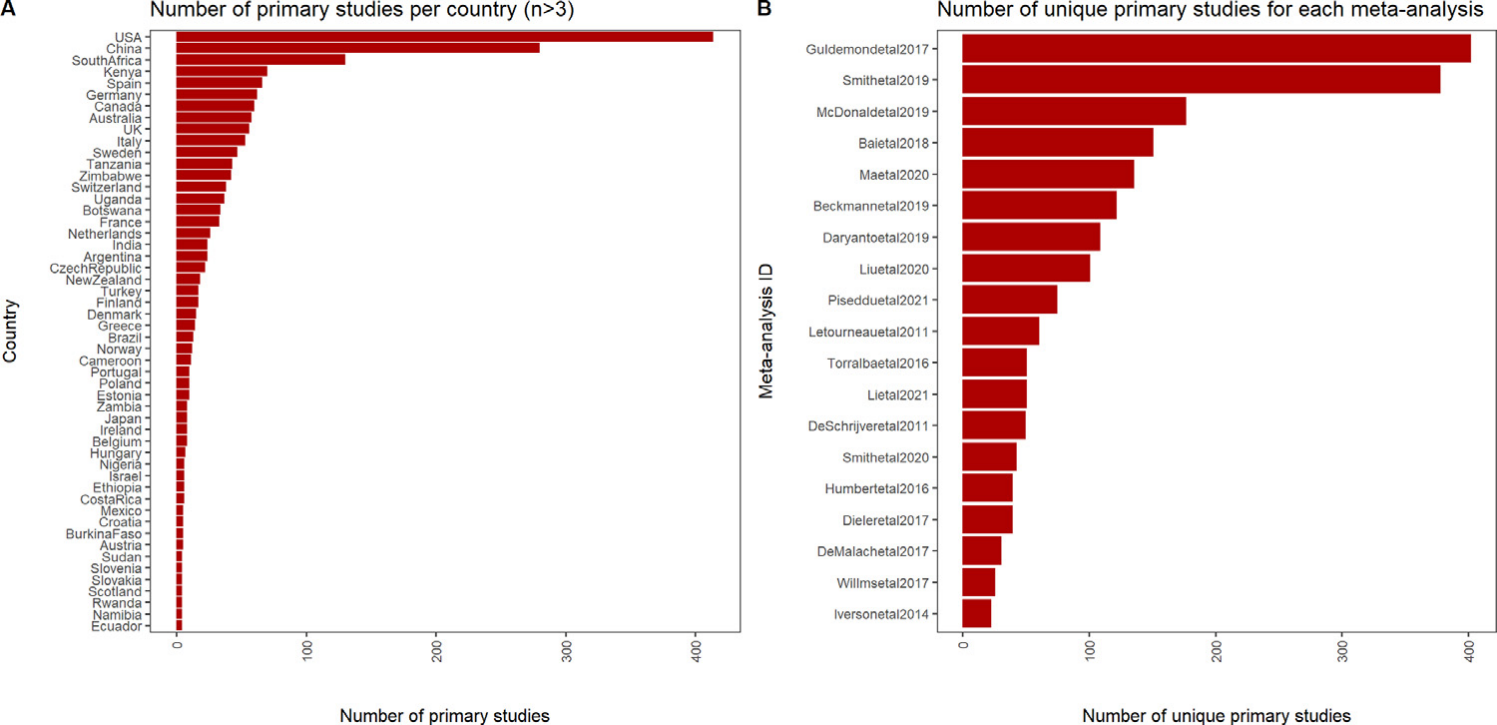
Number of unique primary studies per country (for countries with more than 3 primary studies) (A) and number of unique primary studies of the 31 out of 33 meta-analyses (B) included in the database.

**Fig. 5:**
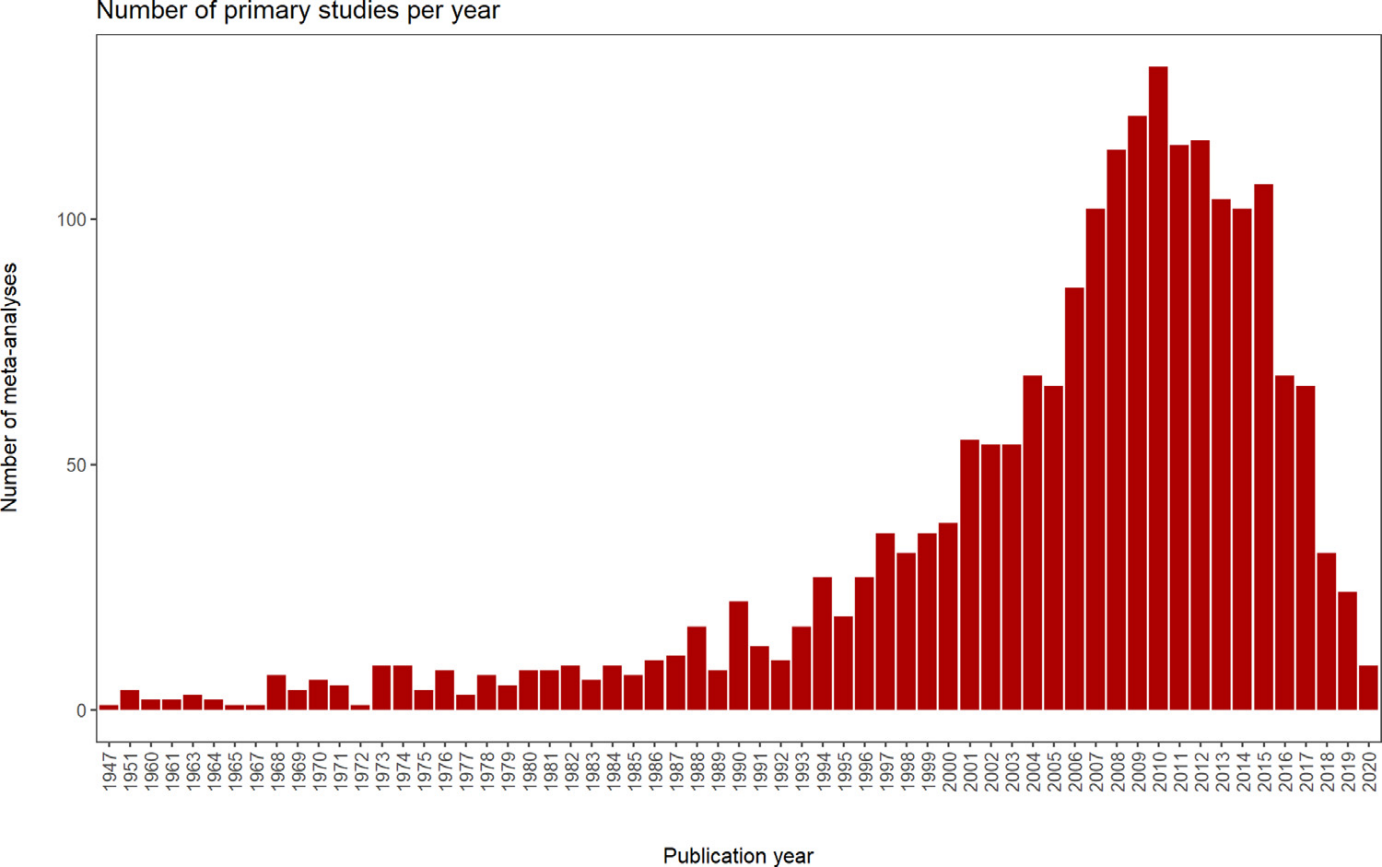
Number of primary studies per year.

The empirical (observational or experimental) papers that are used in a meta-analysis are called primary studies. We compiled a list of all the primary studies that were used in 19 randomly selected studies out of the 33 meta-analyses in our database. From this list, we manually extracted the country (or countries) in which each primary study took place. The final table comprised of 1,967 unique primary studies, that took place in more than 100 countries (Fig. 4), over multiple decades (Fig. 5).

## 3 Experimental Design, Materials and Methods

### 3.1 Description of meta-analyses included in the database

All the meta-analyses in our database were designed by their authors in order to answer the same type of question: “What is the impact of management practice X on (among others) biodiversity and yield?”. Therefore, each meta-analysis includes empirical studies that measure yield and/or biodiversity. At the analysis stage, authors would create subsets for each response variable; one subset for yield and one for biodiversity. These subsets would be then fitted in separate meta-analytic models and meta-regressions (where necessary) with different moderators. The model estimates would be then reported separately for each response variable. Our database consists of those model estimates. In most cases, meta-analyses provided more effect sizes for biodiversity and less for yield, hence we kept the two datasets separate.

### 3.2 Data collection and quality assessment

**Fig. 6:**
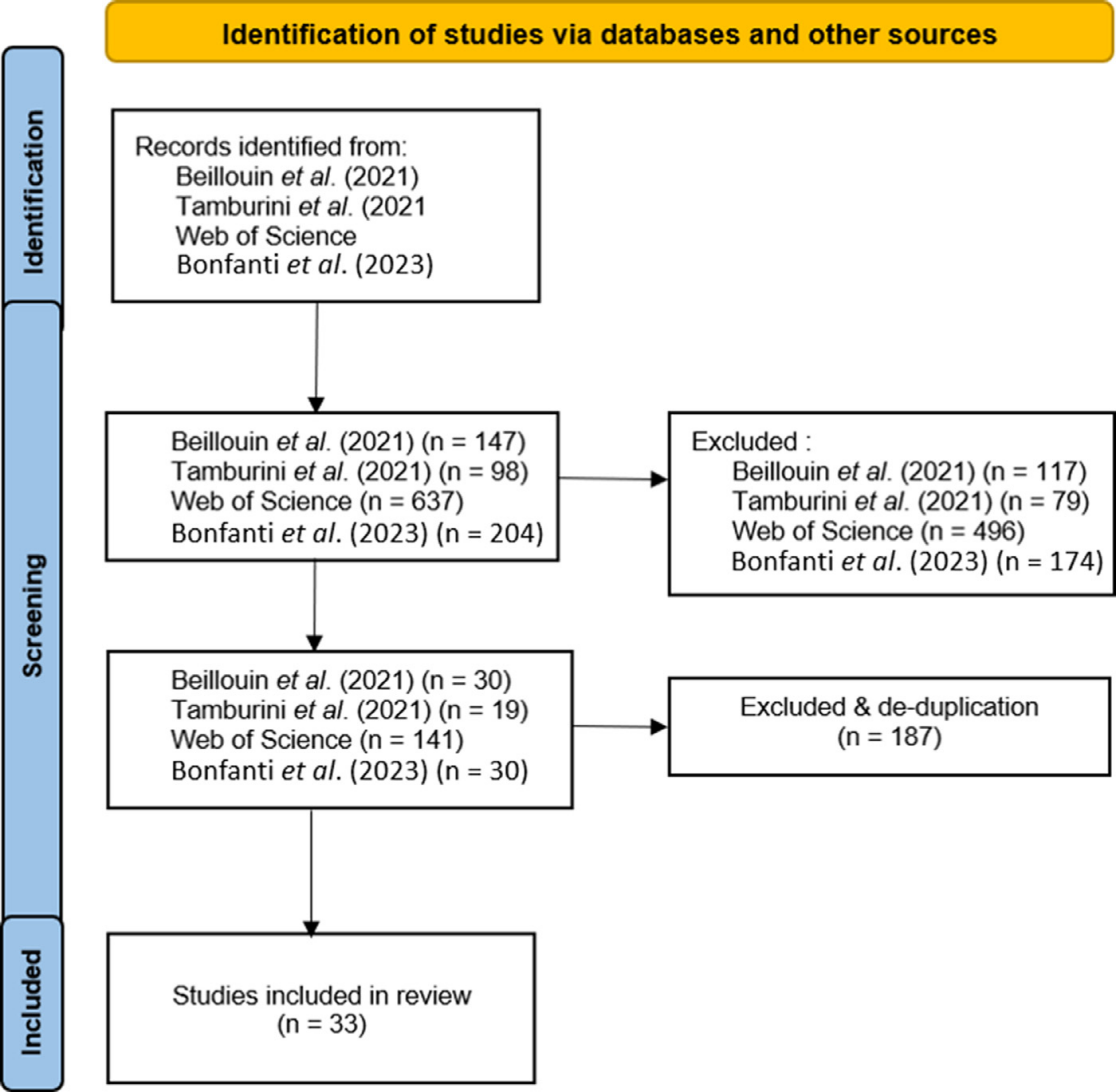
PRISMA diagram. Number of studies that were included and excluded during title, abstract and full-text screening.

Aiming to identify all relevant meta-analyses to build our database, we conducted a systematic literature review according to the PRISMA 2020 guidelines [34]. We used three data sources: i) Web of Science, ii) two second order meta-analyses [35,36] and iii) a database provided by JB [37]. We screened 1,086 titles and abstracts based on the inclusion criteria: i) the study should be a meta-analysis ii) the response variables of the meta-analytic models should be biodiversity and yield iii) the meta-analysis should examine the impact of some agricultural management practice on biodiversity and yield. The same inclusion criteria were used for the full-text screening of 220 publications. After the full-text screening stage, we identified 33 meta-analyses that met the inclusion criteria (Fig. 6). We extracted all effect sizes, effect size metrics, sample sizes, error measures, response variable measures, management practices and study metadata. An effect size is here defined as the meta-analytic model estimate of the effect of a management practice on biodiversity or yield.

In 17 cases, we extracted the effect sizes from figures and plots, using the R package metaDigitise and the application shinyDigitise [38], as well as the online tool WebPlotDigitizer [39]. In the cases where a confidence interval was not provided in the text or in a table, we extracted the standard error from figures using the tools mentioned above and then converted it to a 95 % confidence interval using a customized function, provided in the R script that is located in the OSF folder. We used R software [40] (version 4.3.0) for all figures and plots shown here (ggplot2 3.4.2 [41], dplyr 1.1.2 [42].

**Fig. 7:**
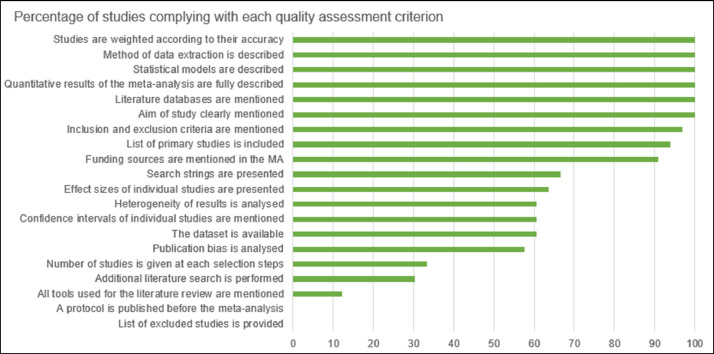
Percentage of studies meeting each quality assessment criterion.

During data extraction, we aggregated raw data in meaningful categories. For example, we aggregated the management practices in 14 levels. Organismal groups were aggregated at the level of taxon. In addition, we compiled all the primary studies from each meta-analysis, wherever possible. We manually extracted the location of each primary study took place, by reading the title, the abstract or the full-text. We evaluated the quality of each meta-analysis in our database using an assessment tool that is specific to meta-analyses in agronomy ([43], as implemented in [44]). This tool includes multiple criteria relative to the methodology of meta-analyses (Fig. 7). After reading each full text, we indicated in the table “quality_assessment_OSF.xlsx” (available in OSF) whether the study complies with each criterion.

## Limitations

Not applicable.

## Ethics Statement

The authors have read and follow the ethical requirements for publication in Data in Brief and confirm that the current work does not involve human subjects, animal experiments, or any data collected from social media platforms.

## CRediT authorship contribution statement

**Elina Takola:** Conceptualization, Methodology, Data curation, Writing – original draft, Visualization, Investigation. **Jonathan Bonfanti:** Data curation, Validation, Writing – review & editing. **Ralf Seppelt:** Conceptualization, Methodology, Writing – review & editing. **Michael Beckmann:** Conceptualization, Methodology, Writing – review & editing.

## Data Availability

An open-access global dataset of meta-analyses investigating yield and biodiversity responses to different management practices. (Reference data) (Open Science Framework (OSF))
